# Barriers to antigenic escape by pathogens: trade-off between reproductive rate and antigenic mutability

**DOI:** 10.1186/1471-2148-7-229

**Published:** 2007-11-15

**Authors:** Steven A Frank, Robin M Bush

**Affiliations:** 1 Department of Ecology and Evolutionary Biology, University of California, Irvine, CA 92697-2525, USA

## Abstract

**Background:**

A single measles vaccination provides lifelong protection. No antigenic variants that escape immunity have been observed. By contrast, influenza continually evolves new antigenic variants, and the vaccine has to be updated frequently with new strains. Both measles and influenza are RNA viruses with high mutation rates, so the mutation rate alone cannot explain the differences in antigenic variability.

**Results:**

We develop a new hypothesis to explain antigenic stasis versus change. We first note that the antigenically static viruses tend to have high reproductive rates and to concentrate infection in children, whereas antigenically variable viruses such as influenza tend to spread more widely across age classes. We argue that, for pathogens in a naive host population that spread more rapidly in younger individuals than in older individuals, natural selection weights more heavily a rise in reproductive rate. By contrast, pathogens that spread more readily among older individuals gain more by antigenic escape, so natural selection weights more heavily antigenic mutability.

**Conclusion:**

These divergent selective pressures on reproductive rate and antigenic mutability may explain some of the observed differences between pathogens in age-class bias, reproductive rate, and antigenic variation.

## Background

Measles vaccines, first deployed in the 1960s, remain effective today. No antigenic variants have spread that escape from immunity against the original Edmonston strain isolated in 1954 and the subsequent vaccine strains derived from that original isolate [1]. By contrast, the influenza vaccine must be updated annually to track the new influenza variants that frequently arise [2]. Among influenza viruses, types A and B are the ones that mutate continuously to produce immune escape variants that cause new epidemics. Influenza type C, however, seems more like measles in that it does not generate variants that escape the immune memory of hosts [3].

Why do some pathogens fail to produce new antigenic variants that escape widespread immunity by the host population, whereas others readily mutate to variants that escape host immunity? This is an important question, because long-term control by vaccination depends on whether immune escape variants arise and, if they do, how quickly such variants appear.

Three factors may explain the rate at which immune escape variants arise and spread. First, some pathogens may have higher mutation rates and thereby generate new variants at a faster rate. This argument particularly applies to the contrast between RNA and DNA viruses, because RNA viruses have mutation rates about two orders of magnitude greater than DNA viruses [4]. Some of the most rapidly evolving viruses are indeed RNA viruses, such as HIV and influenza types A and B. However, measles is also an RNA virus, as is influenza C, the mumps virus, and the rubella virus, all of which lack antigenic variation [1,3,5,6]. So mutation rate cannot be the primary factor.

Second, some pathogens may induce broad immune responses, in which host immunity simultaneously attacks multiple sites on the pathogen. Immune protection against multiple pathogen epitopes may prevent pathogens from generating the multiple mutations required to escape immune recognition [7]. By contrast, when the immune response focuses on only one or a few sites on the pathogen surface, then small changes can allow the pathogen to escape host immunity. This idea probably does explain some of the differences between pathogens. However, there is little direct evidence that relates degree of multisite cross-reactivity of host immunity to the rate at which natural populations of pathogens generate escape variants.

Third, structural constraints may make it difficult for some pathogens to alter their surface molecules at the sites attacked by host antibodies. If the pathogen cannot alter the sites recognized by the host, it cannot generate escape variants. Often, the primary sites of antibody recognition are near the main sites used by the pathogen to attach to host cells [7-9]. For example, it might be that the primary receptor binding protein of the measles virus cannot be altered without significantly reducing the ability of the virus to attach to and enter host cells, whereas influenza types A and B can vary their receptor binding proteins in a way that allows escape from antibodies but preserves binding to cellular receptors.

The hypothesis of structural constraint implies that, if an escape variant arose, its fitness would be so greatly reduced that it could not spread. That explanation raises a key question: how large must the reduction in fitness be to prevent an escape variant from spreading?

The structural constraint argument also raises the problem of why influenza C might face a more severe constraint than do types A and B. The primary host attachment (receptor binding) protein of influenza C differs significantly from that of types A and B [10]. Perhaps there is some structural aspect of attachment in influenza C that limits variation relative to the other types. However, influenza C has been studied relatively little – further study of type C compared with types A and B may provide insight into the causes of antigenic variation.

Overall, the puzzle remains: Why do some pathogens escape frequently and evolve rapidly, whereas others rarely generate escape variants and remain controlled over many years by the same vaccine? In this paper, our primary goal is to frame the problem clearly so as to encourage direct study based on explicit hypotheses. In our quantitative models, we clarify the notion of structural constraint by quantifying how large must be a disadvantage for altering structure in order to outweigh the large benefit of escaping host immunity. We also consider how short-term cross reactivity affects the rise of escape variants, and we quantify how nonequilibrium perturbations and periodic epidemic cycles alter the costs and benefits of escape variants.

After outlining the basic problem and quantifying the main processes, we turn to our own novel hypothesis. We argue that some pathogens concentrate transmission in younger hosts who lack immunity against any variants. Those pathogens compete solely on the basis of rate of infection and transmission rather than on escape from host immunity. In this case, the pathogen variant with the highest reproductive rate dominates over time, with little tendency for evolutionary change by immunological escape.

If a trade-off occurs between reproductive rate and the ability to tolerate the amino acid substitutions needed to escape host immunity, then, over time, selection pressure primarily for highest reproductive rate in naive hosts may cause the pathogen to lose the ability to vary its antigens. By contrast, pathogens that transmit more widely among older hosts, who potentially carry immune memory of past infections, are more likely to evolve escape variants that spread to high frequency, and to evolve greater tolerance to amino acid substitutions (greater mutability) in key antigenic regions of surface molecules.

Measles, mumps, and rubella do concentrate transmission among younger hosts [5,6,11], whereas influenza types A and B spread more widely among different age and exposure classes [12]. These patterns match the predictions of our theory: concentration in the naive classes correlates with lack of antigenic variation. However, this observed correlation leaves open the problem of cause and effect. Does competition primarily in the naive classes favor antigenic dominance by a single type, as our theory predicts? Or does some other cause restrain antigenic variation and therefore restrict infections to the naive class? Our formulation in this paper calls attention to these important questions and frames the problem with clear hypotheses that provide a way forward.

## Results and Discussion

### Previous theories

Keeling [13] provided an interesting theoretical discussion of the trade-off between competition and persistence in pathogens. Here, "persistence" means the tendency of a genotype to resist extinction over the long run. Conceptually, our idea is similar to Keeling's in that both theories consider the alternative selection pressures of immediate reproductive rate versus long-term persistence. In our case, antigenic mutability facilitates long-term persistence, whereas Keeling did not discuss antigenic variation but instead focused on how decreased pathogen virulence increases persistence. The tradeoff between reproductive rate and aspects of persistence also calls to mind the tradeoff between *r *and *K *selection discussed by MacArthur and Wilson [14].

Novella et al. [15] discussed a different trade-off between reproductive rate and antigenic escape. They began with the observation that, in the laboratory, escape variants often suffer a reduced reproductive rate compared with the original strain. When such variants are then grown in the absence of immune pressure, some viral species regain full reproductive potential whereas others do not. Thus, viruses that can escape by antigenic variation and then subsequently recover full reproductive potential may be the ones that show wide antigenic variation in natural populations. By contrast, those viruses that lose adaptability during antigenic escape, and cannot subsequently recover full reproductive potential, are the ones that tend to lack antigenic variation.

Our theory suggests that antigenically variable pathogens may, in fact, be those that have given up the ability to recover full reproductive rate in the absence of immune pressure, in return for the ability to tolerate substitutions that escape immunity. We also argue that those pathogens concentrated in young or immunologically naive hosts would be unlikely to tolerate the cost and be unvarying, whereas those pathogens that spread more widely would tolerate the cost and be antigenically variable.

### Summary of the quantitative models

We seek conditions under which an antigenic variant remains, over time, the dominant type in the pathogen population. By "dominant type" we mean "maintains a greater relative frequency over time." To analyze the conditions for dominance, we compare a potentially dominant type in relation to an alternative variant. Thus, we study a model in which two different antigenic variants of the pathogen exist: type 1 and type 2. We assume that pathogen type 1 has a transmission success greater than or equal to that of type 2. We analyze conditions under which the initially dominant type 1 can remain dominant in the population against subordinate variants of type 2 that have lower transmission rates. The subordinate types, by being rare, have the advantage that fewer hosts carry immunological memory against them. We show below that this advantage of rarity for type 2 often allows the subordinate type to rise in frequency in spite of its transmission disadvantage. The key problem for long-term dominance by a single antigenic variant concerns the balance of the transmission advantage for type 1 against the rarity advantage for type 2. We have defined type 1 as the resident type – this labeling for the initially dominant type is just a convention. We have defined type 2 as a rare variant with a lower reproductive rate (*R*_0_) than type 1. Obviously, if type 2 was a rare variant with higher *R*_0_, then type 2 would invade, and there would be no point in studying invasion conditions. Thus, we can think of type 1 as the highest *R*_0 _variant that could reasonably evolve, and we proceed to study whether type 1 will hold on as the dominant type.

After introducing the model in the next section, we analyze in four steps the conditions for long-term antigenic dominance. First, we quantify the conditions under which a rare type with lower transmission rate can invade against a dominant type with higher transmission rate. Invasion by the rare type requires that the benefit gained by the rare type, through its ability to attack hosts who have immune memory against the common type, outweighs its transmission disadvantage. Although this condition is very simple, it provides significant insight by showing how large a transmission disadvantage must be to prevent invasion by a new, rare antigenic variant.

Second, we show that when a rare type can invade, it almost always increases to a significant frequency, breaking the strong dominance by the original type. Thus, the very simple invasion criteria of the first section provide much insight into the conditions under which antigenic dominance can be maintained. However, these conclusions depend on the assumption that the frequencies of the antigenic variants approach their long-term equilibrium without perturbations that induce nonequilibrium dynamics. 

Third, we analyze the dynamics of invasion to set the stage for understanding the consequences of nonequilibrium perturbations. We study the dynamics of invasion by introducing both types of pathogen into a naive host population with no immunological memory against prior infections. The dominant type increases initially, because the rare type suffers a transmission cost and, at first, all hosts lack immunological memory of prior infections. After a while, immunological memory against the common type builds, providing the rare type with a benefit that offsets its transmission cost, allowing the rare type to rise to a significant frequency.

If epidemics occur in relatively brief pulses, followed by times of low infection, then a rare type that could potentially rise to high abundance over the long run may in fact never increase, because each epidemic pulse in a host population with little immunological memory initially favors the dominant type. The effective immunological memory of host populations may decay quickly if most infections tend to occur among newborns or young, immunologically naive individuals. Those naive individuals tend to be infected in each epidemic cycle, but are soon replaced by recruitment of more newborns, replenishing the supply of immunologically naive individuals. With most infections in those naive hosts, the relative transmission rates of the pathogens rather than immunological memory determine the outcome, favoring the dominant type with the higher transmission rate.

Fourth, we continue study of nonequilibrium dynamics by analyzing epidemics that follow periodic cycles. We argue that periodicity may be driven, in part, by seasonal changes in transmission rate. Periodic fluctuations in transmissibility influence antigenic dominance in ways that make it more difficult for a rare type to invade. Immune memory decays between epidemics, favoring the dominant type as described by the third point above. In addition, with fluctuating transmissibility, the average transmissibility over the whole cycle may decline, and, as we show, lower transmissibility makes invasion by a rare type more difficult. Throughout, when we discuss "antigenic escape," we mean variants that escape from host immune memory in natural populations; we do not discuss the monoclonal escape variants that are often generated in the laboratory [16-18].

Later in the paper, we discuss a tradeoff between *R*_0 _and antigenic mutability. But in the model formulation, we do not explicitly study such a tradeoff. Instead, we simply examine the conditions under which type 1 resists invasion by a rare, lower *R*_0 _type 2, and the conditions under which the rare, lower *R*_0 _type 2 invades the higher *R*_0 _type 1.

Such invasion criteria provide the first step in analyzing the tradeoff between *R*_0 _and antigenic mutability. In particular, if type 1 resists invasion, then types with lower *R*_0 _and higher antigenic mutability will not spread, and we have our answer with regard to how that tradeoff will play out: the highest *R*_0 _type will win. By contrast, if type 2 invades, then there is scope for types with lower *R*_0 _and higher antigenic mutability to succeed. Our mathematical analysis takes us only that far. In cases in which type 2 invades, we do not analyze in detail how the tradeoff between *R*_0 _and antigenic mutability would play out. The details of such a tradeoff would require more complicated mathematical analysis, overwhelming the simplicity of our current formulation. More importantly, there is at present little biological evidence to tell us how to formulate such a tradeoff in a mathematical model. We have therefore chosen to frame the problem simply and clearly, to emphasize the potential for future empirical and theoretical studies.

### Summary of the key parameters

This section emphasizes the biological interpretation of the key parameters. The Methods describes the full model.

We follow three classes of host: *S*, *I*, and *R*. Uninfected hosts of type *S *have been infected previously by a subset of the antigenically variable pathogens. Hosts in the *S *class retain immunological memory against and resist reinfection by those pathogen types that have previously infected them. For example, *S*_1 _resists infection by pathogen type 1 and is susceptible to pathogen type 2, whereas *S*_3 _resists infection by both type 1 and type 2. The naive class *S*_0 _has not previously been infected and is susceptible to both pathogen types.

Infected hosts of type *I *have immunological memory from prior infections and are currently infected by a pathogen with a type not previously encountered. For example, *I*_1,2 _denotes hosts previously infected by type 1 and currently infected by type 2. We denote hosts who currently are infected by type *k *and do not have memory against a prior exposure as *I*_0,*k*_.

Recovered hosts of type *R*_*j *_remain temporarily immune against any new infection because of short-term nonspecific immunity that developed during a recent infection. The subscript *j *encodes the immunological memory of all prior infections, including the most recent infection. After nonspecific immunity decays, hosts of type *R*_*j *_change into hosts of type *S*_*j*_.

We focus on five key parameters. For all parameters, nondimensional time units measure average lifespan. In our formulation, lifespan means either death of an adult and recruitment of a newborn into the naive class, *S*_0_, or loss of immunological memory that reassigns a previously infected adult to the naive class (see Methods).

First, *α *is the rate at which a host clears an infection: the transition from *I *to *R*. Second, *β *is the rate at which the dominant type 1 pathogens infect a susceptible individual who has not previously been infected: the transition from *S *to *I*. Third, the transmission parameter for the subordinate type 2 pathogens is *β*(1 - *δ*/*β*). Here, *δ*/*β *measures the cost to pathogen type 2 for evolving the ability to escape from the host's immune response against type 1. Fourth, *c *is the rate at which a recovered and nonspecifically immune host becomes susceptible to infection by antigenic types for which it has no prior history of infection: the transition from *R *to *S*.

A fifth factor, *γ*, also influences transmission. The naive class of hosts, with no memory against prior infections, often corresponds to newborns or younger individuals who have not yet been exposed to infection, whereas the classes with memory of prior exposure often correspond to older individuals who have been previously infected. Transmission may occur more rapidly between newborns and younger individuals than between older individuals: the younger classes may aggregate more often in schools or play, or may be inherently weaker and more prone to infection.

We can account for faster transmission among the naive class compared with the exposed classes by reducing the transmission of new infections to those who carry memory of past infections. In particular, we weight transmission to susceptibles who have memory of prior infection by *γ *≤ 1. We vary *γ *to provide a simple method for studying the role of age-related effects on transmission. Technically, our mathematical approach for this factor differs from the standard mathematical methods for studying age structure. In the Methods, we discuss our method in relation to traditional approaches.

In our main analyses of invasion and long-term equilibrium, we look at a wide array of parameter values, so that one can see how the outcome changes over any reasonable assumptions. For our analyses of dynamics, we emphasize qualitative processes that do not depend strongly on particular parameter values.

### Invasion

Immunological memory opposes antigenic dominance: dominant types create a higher frequency of immunological memory in hosts than do rare types, and thus dominant types face more resistance against future infection. A rare type therefore invades and challenges the dominant type, unless the rare type bears a cost sufficient to outweigh its inherent advantage.

In this section, we provide an expression for the benefits and costs of rare variants. That expression provides a condition under which rare types can invade and challenge the dominant type. Studying invasion of rare types does not provide a full analysis of the long-term conditions for antigenic dominance. But, as we show later, such analysis does provide much insight.

To study invasion of a rare type, we first set the abundance of the rare variant (type 2) to zero and solve for the equilibrium state of the host population containing only the candidate dominant type (type 1). We then introduce a rare infection of the second variant (type 2) by setting the total fraction of hosts infected by the rare variant to a small value. As shown in the Methods, the rare type increases from an initially low level when

γS1∗S0∗+γS1∗>δβ,
 MathType@MTEF@5@5@+=feaafiart1ev1aaatCvAUfKttLearuWrP9MDH5MBPbIqV92AaeXatLxBI9gBaebbnrfifHhDYfgasaacPC6xNi=xI8qiVKYPFjYdHaVhbbf9v8qqaqFr0xc9vqFj0dXdbba91qpepeI8k8fiI+fsY=rqGqVepae9pg0db9vqaiVgFr0xfr=xfr=xc9adbaqaaeGacaGaaiaabeqaaeqabiWaaaGcbaqcfa4aaSaaaeaaiiGacqWFZoWzcqWGtbWudaqhaaqaaiabigdaXaqaaiabgEHiQaaaaeaacqWGtbWudaqhaaqaaiabicdaWaqaaiabgEHiQaaacqGHRaWkcqWFZoWzcqWGtbWudaqhaaqaaiabigdaXaqaaiabgEHiQaaaaaGaeyOpa4ZaaSaaaeaacqWF0oazaeaacqWFYoGyaaGaeiilaWcaaa@3FB3@

where the stars denote the equilibrium values when only type 1 is present.

This inequality highlights the relative benefits and costs of the rare type compared with the dominant type. On the left side, the rare type gains by being able to infect those hosts who have memory against the dominant type, S1∗
 MathType@MTEF@5@5@+=feaafiart1ev1aaatCvAUfKttLearuWrP9MDH5MBPbIqV92AaeXatLxBI9gBaebbnrfifHhDYfgasaacPC6xNi=xH8viVGI8Gi=hEeeu0xXdbba9frFj0xb9qqpG0dXdb9aspeI8k8fiI+fsY=rqGqVepae9pg0db9vqaiVgFr0xfr=xfr=xc9adbaqaaeGacaGaaiaabeqaaeqabiWaaaGcbaGaem4uam1aa0baaSqaaiabigdaXaqaaiabgEHiQaaaaaa@2F0E@, with a discount of *γ *for transmission into hosts with memory of prior infection. This transmission advantage for rare types is scaled in the denominator by the total amount of transmission into all types of susceptible hosts. On the right side, the rare type suffers a cost, *δ*/*β*, through its lower transmission efficiency than the dominant type. This cost measures the fractional reduction in transmission of the rare type compared with the common type.

The rare type invades when it gains more through its ability to attack hosts who have immunological memory against the dominant type than it loses by reduced transmission efficiency. Generally, the invasion condition can be satisfied by a rare mutant that does not pay too high a cost in transmission (*δ*/*β *not too high), causing the benefit of attack against those hosts that have memory against the dominant type to outweigh the transmission cost.

**Figure 1:**
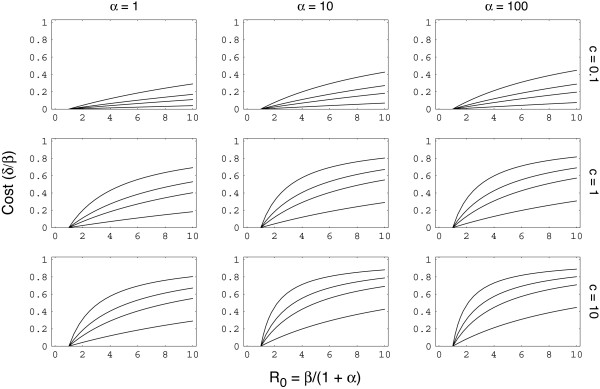
**Threshold for invasion by rare antigenic variant into a population at equilibrium for a dominant variant**. The *y *axis in each plot shows the maximum cost, *δ*/*β*, that an invading variant can carry and still succeed in invasion; lower values correspond to more stringent conditions for invasion by a rare type and greater scope for dominance by the common type. The *x *axis shows *R*_0 _= *β*/(1 + *α*). *R*_0 _is the basic reproductive number of the pathogen – the average number of new infections caused by a single infection in a fully susceptible host population [23]. The four curves in each panel show values of *γ *= 0.1, 0.3, 0.5, 1.0 from bottom to top. Because of the nondimensional scaling of time used for rate parameters, faster death rate, or decay of memory, reduces all parameters by the same scaling factor. The consequences of a greater death rate are shown approximately by moving from the lower right panel to the upper left panel along the diagonal, in which invasion by a rare type becomes more difficult. Faster decay of memory reduces the benefit to the rare type by reducing the relative frequency of *S*_1_, the hosts that carry memory against the dominant type. Thus, hosts with shorter generation times are more likely to support pathogens that maintain antigenic dominance over long periods of time.

Figure 1 shows the criteria for invasion in terms of particular parameter values. The main points can be summarized as follows. Assume that *δ *scales with *β*, so that the cost *δ*/*β *remains fixed as *β *changes. Then a rise in *β *favors invasion by the rare type, because greater intensity of transmission shifts the hosts away from the fully susceptible class, S0∗
 MathType@MTEF@5@5@+=feaafiart1ev1aaatCvAUfKttLearuWrP9MDH5MBPbIqV92AaeXatLxBI9gBaebbnrfifHhDYfgasaacPC6xNi=xH8viVGI8Gi=hEeeu0xXdbba9frFj0xb9qqpG0dXdb9aspeI8k8fiI+fsY=rqGqVepae9pg0db9vqaiVgFr0xfr=xfr=xc9adbaqaaeGacaGaaiaabeqaaeqabiWaaaGcbaGaem4uam1aa0baaSqaaiabicdaWaqaaiabgEHiQaaaaaa@2F0C@, and toward the recovered class, S1∗
 MathType@MTEF@5@5@+=feaafiart1ev1aaatCvAUfKttLearuWrP9MDH5MBPbIqV92AaeXatLxBI9gBaebbnrfifHhDYfgasaacPC6xNi=xH8viVGI8Gi=hEeeu0xXdbba9frFj0xb9qqpG0dXdb9aspeI8k8fiI+fsY=rqGqVepae9pg0db9vqaiVgFr0xfr=xfr=xc9adbaqaaeGacaGaaiaabeqaaeqabiWaaaGcbaGaem4uam1aa0baaSqaaiabigdaXaqaaiabgEHiQaaaaaa@2F0E@, which can be attacked only by the rare type. Similarly, for reasonable parameter values, rises in *α *and *c *favor invasion because both parameters enhance movement of individuals into S1∗
 MathType@MTEF@5@5@+=feaafiart1ev1aaatCvAUfKttLearuWrP9MDH5MBPbIqV92AaeXatLxBI9gBaebbnrfifHhDYfgasaacPC6xNi=xH8viVGI8Gi=hEeeu0xXdbba9frFj0xb9qqpG0dXdb9aspeI8k8fiI+fsY=rqGqVepae9pg0db9vqaiVgFr0xfr=xfr=xc9adbaqaaeGacaGaaiaabeqaaeqabiWaaaGcbaGaem4uam1aa0baaSqaaiabigdaXaqaaiabgEHiQaaaaaa@2F0E@, the host class with memory and protection against the dominant type but not against the rare invader.

Finally, a decline in *γ *reduces the intensity of transmission by the rare type into S1∗
 MathType@MTEF@5@5@+=feaafiart1ev1aaatCvAUfKttLearuWrP9MDH5MBPbIqV92AaeXatLxBI9gBaebbnrfifHhDYfgasaacPC6xNi=xH8viVGI8Gi=hEeeu0xXdbba9frFj0xb9qqpG0dXdb9aspeI8k8fiI+fsY=rqGqVepae9pg0db9vqaiVgFr0xfr=xfr=xc9adbaqaaeGacaGaaiaabeqaaeqabiWaaaGcbaGaem4uam1aa0baaSqaaiabigdaXaqaaiabgEHiQaaaaaa@2F0E@, the host class that carries memory against the dominant type. A sufficient transmission discount (lower *γ*) can prevent invasion by a rare antigenic type.

Relations similar to Eq. (1) often arise in the literature on mathematical epidemiology, in which models interpret *γ *as a measure of cross reactivity between different antigenic variants (Abu-Raddad and Ferguson [19] provide a good summary of the different mathematical models of cross reactivity). In our model, *γ *gives a simple way to account for the potentially higher rates of transmission that may occur among younger hosts. The Methods summarizes technical issues in regard to the interpretation of *γ*.

### Equilibrium

In this section, we show that when a rare type does invade, it almost always increases to a fairly high level, breaking the dominance by the original type. Thus, the criteria for invasion in the prior section provide a good guide to the conditions for long-term dominance when no extrinsic factors perturb the system. To measure long-term dominance, we calculated the long-term equilibrium of the dominant and rare types when both types are present. We measure dominance by the ratio of the original type to the invading type at equilibrium. Numerical analysis suggests that the equilibrium for the system in Eqs. (2–12) of the Methods is neutrally stable, but our analyses also show that the system, when far away from equilibrium, does tend to flow toward the neighborhood of the equilibrium.

**Figure 2:**
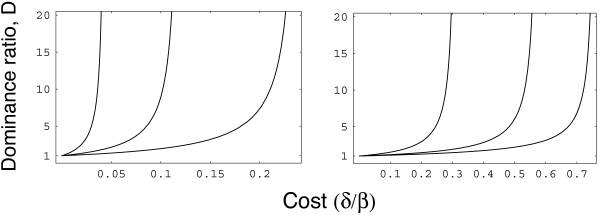
**Equilibrium abundance of the common pathogen type relative to the subordinate variant**. The *y *axis shows the dominance ratio, *D*: the abundance of the common type divided by the abundance of the rare type. In general, the skew in abundances remains moderate until the parameters move near the boundary conditions for invasion shown in Figure 1. Near the boundary, the abundance of the rare type drops sharply, causing a steep rise in relative dominance by the common type. The *x *axis shows the cost, *δ*/*β*, for the rare type. The three curves in each panel show, from left to right, the values for *R*_0 _= 2, 4, 8, which implies that *β *= *k*(1 + *α*) for *k *= 2, 4, 8. We used the parameters *α *= 10 and *c *= 1 to match the center panel of Figure 1. The left panel shows *γ *= 0.1, and the right panel shows *γ *= 1.

Figure 2 illustrates the sharp change in the dominance ratio near the invasion threshold given by Eq. (1). The *x *axis shows the cost for the rare type, and the *y *axis shows the dominance ratio. A cost above the invasion threshold means that the rare type cannot invade. As the cost declines slightly from the invasion threshold, allowing the rare type to invade, the long-term equilibrium dominance by the original type drops quickly. Thus, a cost above the invasion threshold causes total dominance by the primary type; a cost slightly below the invasion threshold allows the rare type to rise to relatively high frequency, breaking the dominance of the original type.

These results about the long-term equilibrium with both types present show that the invasion criteria in Eq. (1) provide a good guide to whether the initial type can maintain antigenic dominance at a long-term equilibrium.

### Dynamics

To calculate the invasion criteria for the rare type in Eq. (1), we first set the system at equilibrium with only the dominant type present. We then examined the conditions under which a rare variant could invade. To analyze dominance at a long-term equilibrium, we calculated the equilibrium when both pathogen types are present and eventually settle near their long-term abundances in the host population.

Many infectious diseases never settle to an equilibrium. Epidemics may follow a periodic cycle set by the seasons, as in influenza; epidemics may follow a period set by the yearly recruitment of newborn hosts, as sometimes happens in measles; or epidemics may arise sporadically.

Recurrent perturbations away from equilibrium can shift the criteria for dominance by a single antigenic variant. For example, a cycle in which infections become rare following each epidemic may prevent the maintenance of hosts with immunological memory. Lack of memory works against rare invaders, which gain their advantage by facing less immunological memory than the common type.

To study the role of dynamics, we analyze in this section how the frequencies of the dominant type and the rare type both change over time when they are simultaneously introduced into a naive host population with no immunological memory. At introduction, type 1 is common and type 2 is rare. We then follow the subsequent dynamics.

**Figure 3:**
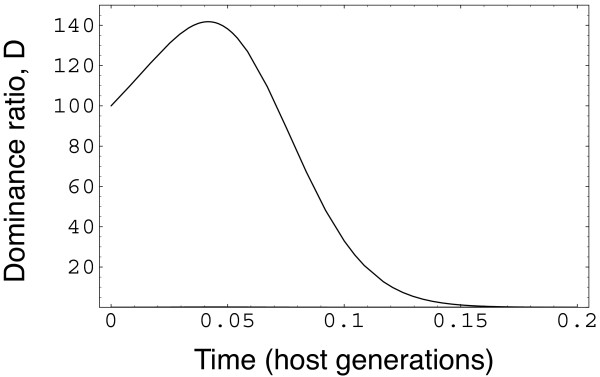
**Dynamics of dominance by the common type**. The dominance ratio, *D*, on the *y *axis is the abundance of the common type divided by the abundance of the rare type, *D *= *I*_·1_*/I*_·2 _(see Appendix). Initially, *I*_01 _= 0.01, *I*_02 _= 0.0001, and *S*_0 _= 0.99. Time is measured by the average lifespan of a host. Parameters are the same as the lower right panel of Figure 1, with *R*_0 _= 2, *γ *= 1, and the transmission cost for the rare type of *δ*/*β *= 0.06.

Figure 3 measures the relative dominance of the common type, given as the ratio of the abundance of the common type to the rare type on the *y *axis. The *x *axis measures time in units of host generations. In this example, the dominant type 1 pathogen at first increases, then the trend reverses, and the rare type 2 pathogen rises in relative abundance.

The dominant type increases initially, because the rare type suffers a transmission cost and, at first, all hosts lack immunological memory of prior infections. After a while, immunological memory against the common type builds, providing the rare type with a benefit that offsets its transmission cost. The dominance ratio quickly falls toward its equilibrium value of 1.04.

Suppose epidemics occur in relatively brief pulses, followed by times of low infection. A rare type that could potentially rise to high abundance over the long run may in fact never increase, because each epidemic pulse in a host population with little immunological memory initially favors the dominant type, as in the early phase of the dynamics in Figure 3.

To prevent the rare type from rising to high abundance, the waiting time between epidemic pulses must be sufficient to reduce host immune memory and the benefit to the invading type. If most infections occur among the naive class (small value of *γ*), then the effective immune memory of the host population will decay quickly, because the naive class is constantly replenished with newborns or, perhaps, with individuals who have lost immunological memory to prior infections.

Thus, a relatively short time between epidemic pulses may be sufficient to favor long-term antigenic dominance by the pathogen type with the highest transmission rate in a naive host population. By contrast, without nonequilibrium perturbations, a rare type with a lower transmission rate can often rise to high abundance, breaking the antigenic dominance by a type with a higher transmission rate in naive host populations.

### Periodic epidemics

In the previous section, we emphasized how nonequilibrium perturbations can favor the dominant type by reducing the amount of immunological memory in the host population. In this section, we study how another aspect of dynamics can also work against rare types and favor long-term antigenic dominance by the type with the highest transmission rate in naive host populations.

Many infectious diseases cause periodic epidemics. Epidemic peaks often occur in particular seasons, such as the annual influenza epidemics of the winter months. The causes of seasonality are not fully understood [20]. One possibility is that host contacts or viral transmissibility per contact rise during certain times of the year and fall during other times of the year. We analyze periodic epidemics by setting the transmissibility parameter to rise and fall in a periodic way.

Periodic fluctuations in transmissibility have two effects on antigenic dominance, both of which make it more difficult for a rare type to invade. First, population-wide immune memory decays between epidemics because of newborns and perhaps memory decay within individuals, favoring the dominant type in the way described in the prior section. Second, with fluctuating transmissibility, the average transmissibility over the whole cycle may decline: lower transmissibility makes invasion by a rare type more difficult (Figure 1). We measure transmissibility by *R*_0 _= *β*/(1 + *α*), where *β *is transmissibility and *α *is the clearance rate of infections; we measure the invasion criteria by the maximum cost in transmissibility that the invading type can sustain and still succeed in invasion (Figure 1).

To study an example of fluctuating transmissibility, suppose that *p *epidemics occur over the time of a typical host lifespan. Then an epidemic peak happens every 1/*p *time units, where time is measured in lifespan. This periodicity may happen in various ways, such as in a host that typically lives *p *years and suffers an annual epidemic, each year comprising a fraction 1/*p *of the lifespan.

To model epidemics with interpeak times of 1/*p*, we scale all transmission rates by a periodic function *σ*(*τ*), where this scaling factor varies between zero and one. Thus, the parameter *β *becomes the transmissibility of the dominant type at the epidemic peak, with actual transmissibility varying according to *σ*(*τ*)*β*. For the rare type, transmissibility becomes *σ*(*τ*)*β*(1 - *δ*/*β*).

**Figure 4:**
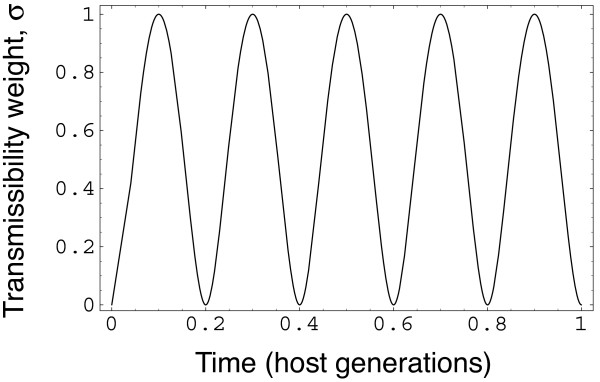
**Periodicity of epidemics driven by fluctuations in transmissibility**. The figure plots the function *σ*(*τ*) = 2^2*a*^*x*^*a*^(1 - *x*)^*a*^, with *p *= 5 and *a *= 2, where *τ *measures time in host generations. The value *x *= *pτ *- floor(*pτ*), where floor(*pτ*) is the largest integer less than or equal to *pτ*. This definition causes *x *to increase linearly from zero to one and then be reset to zero, with *p *cycles in each time unit. We use this function for *σ*, which is a modified form of the beta probability distribution function, to provide a convenient form of cycling for numerical analysis. We did not use sinusoidal functions for seasonality, because those functions drop too sharply at the end of seasons and rise too rapidly at the start of seasons, rather than having more realistically shaped long, flat interseaonal periods as described by the functions that we used.

**Figure 5:**
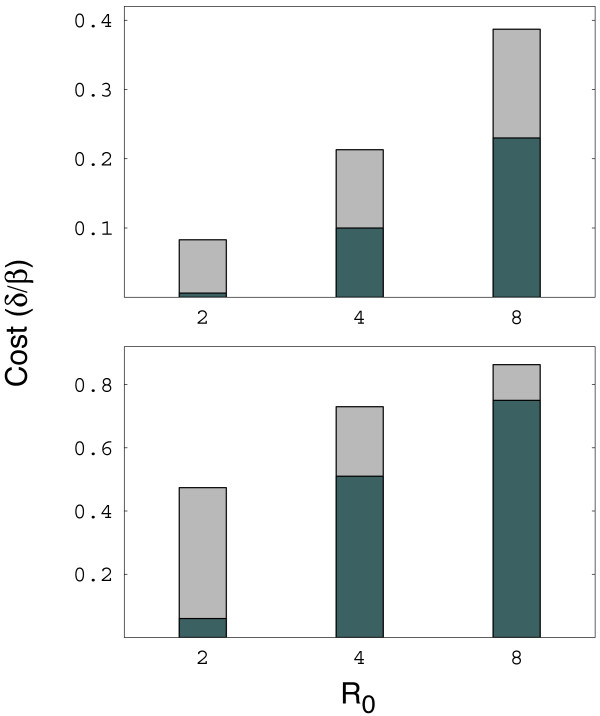
**Periodic epidemics increase the tendency for dominant types to resist invasion by rare types**. The height of each bar shows the maximum cost that a rare type can bear and still successfully invade a dominant type. The light bars show the maximum cost when there is no periodicity in epidemics; we took those values from the lower right panel of Figure 1. The lower values of the dark bars show the more stringent conditions for invasion under periodic epidemics, in which rare types can invade only when they carry relatively lower costs than under nonperiodic epidemics. The top panel shows *γ *= 0.1; the lower panel shows *γ *= 1. For the periodic cases, *p *= 20 and *a *= 2. All other parameters match the lower right panel of Figure 1.

Figure 4 shows an example of how to analyze periodicity in σ, and plots a case with five periods in each lifespan, *p *= 5. Figure 5 shows that periodicity can greatly increase the tendency for dominant types to resist invasion. Thus, periodic epidemic cycles may favor long-term antigenic dominance.

## Conclusion

A rare antigenic variant escapes the immune memory of the host population. That rare-type advantage is powerful: a new variant would typically spread to high frequency even if its reproductive potential (*R*_0_) is greatly reduced by the mutations that allow it to escape host immunity. Figure 1 quantifies the percent reduction in *R*_0 _that an invader may suffer and still successfully increase in frequency. For many realistic parameter combinations, a reduction of 50% easily allows spread of the rare type; in several cases, a rare invading type can suffer an 80% reduction in *R*_0 _and still spread to high frequency.

Several common RNA viruses do not vary antigenically, such as measles, mumps, rubella, and influenza C [1,3,5,6]. What constrains the spread of a variant? It may be that an escape variant never occurs without essentially destroying the capacity to cause an epidemic. Such limitation may arise from structural constraint. Or perhaps a variant could occur, but the probability of the necessary mutational combination is so low that it has not yet been seen. One cannot, at present, rule out either explanation, but given the high mutation rates and vast population sizes of these viruses, the lack of escape variants is interesting. Given the high antigenic mutability of some RNA viruses, such as influenza A and B, HIV, and foot-and mouth-disease virus, why are measles, mumps, rubella, and influenza C so constrained? Is it just luck, or is there some aspect of transmission and epidemiology that leads to constrained versus mutable pathogens? Here, "mutable" means the ability to vary antigenically without great loss in infectivity or transmissibility. In this paper, we have focused on epidemiological factors that seem to separate antigenically constrained from antigenically mutable pathogens. The antigenically constrained RNA viruses are childhood diseases with high *R*_0_, whereas the antigenically mutable viruses spread more readily across age classes.

We showed, with our mathematical models, that a trade-off exists between higher *R*_0 _and antigenic escape from host immunity. Among pathogens that, in a naive host population, spread more rapidly in younger individuals than in older individuals (low *γ*), natural selection weights more heavily a rise in *R*_0_. By contrast, pathogens that spread more readily among older individuals gain more by antigenic escape, so natural selection weights more heavily antigenic mutability. The divergent selection pressures on *R*_0 _versus mutability become even stronger in pathogens that cause periodic or sporadic epidemics.

Based on this theory, we suggest that the unvarying childhood diseases have been shaped by natural selection to have higher *R*_0 _at the expense of structural flexibility and mutability. By contrast, the diseases that spread more readily among adults in an entirely naive host population have been shaped by natural selection to retain structural flexibility and mutability at the expense of a lower *R*_0 _than could be achieved if selection focused only on reproductive rate.

By our theory, comparison of closely related pathogen species that have diverged along these alternative routes should show a variety of structural and functional differences. The key to testing this theory will be such comparisons between closely related but strategically divergent pathogens.

Along these lines, the contrast between influenza type C and types A and B is particularly interesting. Little is known about influenza C: it seems primarily to infect children and to lack antigenic variation. Two studies suggest that both children and adults suffer repeated reinfection by influenza C [21,22], implying that hosts lose immune memory against this antigenically unvarying virus. If so, then influenza C faces a host population with naive children and some naive adults who have lost their immunity from earlier infections. With influenza C infections concentrated in naive children and adults, selection favors high *R*_0 _rather than high mutability. Here, further study might be able to tease apart cause and effect. How has influenza C changed structurally relative to types A and B? Can one recognize, in type C, greater selective pressure for reproductive rate compared with mutability when contrasted with types A and B?

## Methods

### Dynamics

We seek conditions under which an antigenic variant remains the dominant type in the pathogen population. We can get a good idea of the conditions for dominance by studying a potentially dominant type in relation to an alternative variant. Thus, we study a model in which two different antigenic variants of the pathogen exist: type 1 and type 2.

We follow three classes of host: *S*, *I*, and *R*. Uninfected hosts of type *S*_*j *_have been infected previously by a subset, *j*, of the antigenically variable pathogens. Subscripts take on one of four values, *j *= 0, 1, 2, 3, denoting, respectively, prior infection by no pathogens, by type 1 only, by type 2 only, and by both type 1 and type 2. Hosts in the *S *class retain immunological memory against and resist reinfection by those pathogen types that have previously infected them. For example, *S*_1 _resists infection by pathogen type 1 and is susceptible to pathogen type 2, whereas *S*_3 _resists infection by both type 1 and type 2.

Infected hosts of type *I*_*jk *_have, from prior infections, immunological memory against the pathogens encoded by *j *and are currently infected by a pathogen with a type encoded by *k*. For example, *I*_1,2 _denotes hosts previously infected by type 1 and currently infected by type 2. *I*_·*k *_= ∑_*j*_*I*_*jk *_is the total number of hosts infected by the pathogen type *k*.

Recovered hosts of type *R*_*j *_remain temporarily immune against any new infection because of short-term nonspecific immunity that developed during a recent infection. The subscript *j *encodes the immunological memory of all prior infections, including the most recent infection. After nonspecific immunity decays, hosts of type *R*_*j *_change into hosts of type *S*_*j*_.

The 11 equations for the dynamics of the different host classes are

S˙
 MathType@MTEF@5@5@+=feaafiart1ev1aaatCvAUfKttLearuWrP9MDH5MBPbIqV92AaeXatLxBI9gBaebbnrfifHhDYfgasaacPC6xNi=xI8qiVKYPFjYdHaVhbbf9v8qqaqFr0xc9vqFj0dXdbba91qpepeI8k8fiI+fsY=rqGqVepae9pg0db9vqaiVgFr0xfr=xfr=xc9adbaqaaeGacaGaaiaabeqaaeqabiWaaaGcbaGafm4uamLbaiaaaaa@2D59@_0 _= 1 - *βS*_0_*I*_·1 _- (*β *- *δ*)*S*_0_*I*_·2 _- *S*_0_

S˙
 MathType@MTEF@5@5@+=feaafiart1ev1aaatCvAUfKttLearuWrP9MDH5MBPbIqV92AaeXatLxBI9gBaebbnrfifHhDYfgasaacPC6xNi=xI8qiVKYPFjYdHaVhbbf9v8qqaqFr0xc9vqFj0dXdbba91qpepeI8k8fiI+fsY=rqGqVepae9pg0db9vqaiVgFr0xfr=xfr=xc9adbaqaaeGacaGaaiaabeqaaeqabiWaaaGcbaGafm4uamLbaiaaaaa@2D59@_1 _= -*γ*(*β *- *δ*)*S*_1_*I*_·2 _+ *cR*_1 _- *S*_1_

S˙
 MathType@MTEF@5@5@+=feaafiart1ev1aaatCvAUfKttLearuWrP9MDH5MBPbIqV92AaeXatLxBI9gBaebbnrfifHhDYfgasaacPC6xNi=xI8qiVKYPFjYdHaVhbbf9v8qqaqFr0xc9vqFj0dXdbba91qpepeI8k8fiI+fsY=rqGqVepae9pg0db9vqaiVgFr0xfr=xfr=xc9adbaqaaeGacaGaaiaabeqaaeqabiWaaaGcbaGafm4uamLbaiaaaaa@2D59@_2 _= -*γβS*_2_*I*_·1 _+ *cR*_2 _- *S*_2_

S˙
 MathType@MTEF@5@5@+=feaafiart1ev1aaatCvAUfKttLearuWrP9MDH5MBPbIqV92AaeXatLxBI9gBaebbnrfifHhDYfgasaacPC6xNi=xI8qiVKYPFjYdHaVhbbf9v8qqaqFr0xc9vqFj0dXdbba91qpepeI8k8fiI+fsY=rqGqVepae9pg0db9vqaiVgFr0xfr=xfr=xc9adbaqaaeGacaGaaiaabeqaaeqabiWaaaGcbaGafm4uamLbaiaaaaa@2D59@_3 _= *cR*_3 _- *S*_3_

I˙
 MathType@MTEF@5@5@+=feaafiart1ev1aaatCvAUfKttLearuWrP9MDH5MBPbIqV92AaeXatLxBI9gBaebbnrfifHhDYfgasaacPC6xNi=xI8qiVKYPFjYdHaVhbbf9v8qqaqFr0xc9vqFj0dXdbba91qpepeI8k8fiI+fsY=rqGqVepae9pg0db9vqaiVgFr0xfr=xfr=xc9adbaqaaeGacaGaaiaabeqaaeqabiWaaaGcbaqcfaOafmysaKKbaiaaaaa@2DD3@_01 _= *βS*_0_*I*_·1 _- *αI*_01 _- *I*_01_

I˙
 MathType@MTEF@5@5@+=feaafiart1ev1aaatCvAUfKttLearuWrP9MDH5MBPbIqV92AaeXatLxBI9gBaebbnrfifHhDYfgasaacPC6xNi=xI8qiVKYPFjYdHaVhbbf9v8qqaqFr0xc9vqFj0dXdbba91qpepeI8k8fiI+fsY=rqGqVepae9pg0db9vqaiVgFr0xfr=xfr=xc9adbaqaaeGacaGaaiaabeqaaeqabiWaaaGcbaqcfaOafmysaKKbaiaaaaa@2DD3@_02 _= (*β *- *δ*)*S*_0_*I*_·2 _- *αI*_02 _- *I*_02_

I˙
 MathType@MTEF@5@5@+=feaafiart1ev1aaatCvAUfKttLearuWrP9MDH5MBPbIqV92AaeXatLxBI9gBaebbnrfifHhDYfgasaacPC6xNi=xI8qiVKYPFjYdHaVhbbf9v8qqaqFr0xc9vqFj0dXdbba91qpepeI8k8fiI+fsY=rqGqVepae9pg0db9vqaiVgFr0xfr=xfr=xc9adbaqaaeGacaGaaiaabeqaaeqabiWaaaGcbaqcfaOafmysaKKbaiaaaaa@2DD3@_12 _= *γ*(*β *- *δ*)*S*_1_*I*_·2 _- *αI*_12 _- *I*_12_

I˙
 MathType@MTEF@5@5@+=feaafiart1ev1aaatCvAUfKttLearuWrP9MDH5MBPbIqV92AaeXatLxBI9gBaebbnrfifHhDYfgasaacPC6xNi=xI8qiVKYPFjYdHaVhbbf9v8qqaqFr0xc9vqFj0dXdbba91qpepeI8k8fiI+fsY=rqGqVepae9pg0db9vqaiVgFr0xfr=xfr=xc9adbaqaaeGacaGaaiaabeqaaeqabiWaaaGcbaqcfaOafmysaKKbaiaaaaa@2DD3@_21 _= *γβS*_2_*I*_·1 _- *αI*_21 _- *I*_21_

R˙
 MathType@MTEF@5@5@+=feaafiart1ev1aaatCvAUfKttLearuWrP9MDH5MBPbIqV92AaeXatLxBI9gBaebbnrfifHhDYfgasaacPC6xNi=xI8qiVKYPFjYdHaVhbbf9v8qqaqFr0xc9vqFj0dXdbba91qpepeI8k8fiI+fsY=rqGqVepae9pg0db9vqaiVgFr0xfr=xfr=xc9adbaqaaeGacaGaaiaabeqaaeqabiWaaaGcbaGafmOuaiLbaiaaaaa@2D57@_1 _= *αI*_01 _- *cR*_1 _- *R*_1_

R˙
 MathType@MTEF@5@5@+=feaafiart1ev1aaatCvAUfKttLearuWrP9MDH5MBPbIqV92AaeXatLxBI9gBaebbnrfifHhDYfgasaacPC6xNi=xI8qiVKYPFjYdHaVhbbf9v8qqaqFr0xc9vqFj0dXdbba91qpepeI8k8fiI+fsY=rqGqVepae9pg0db9vqaiVgFr0xfr=xfr=xc9adbaqaaeGacaGaaiaabeqaaeqabiWaaaGcbaGafmOuaiLbaiaaaaa@2D57@_2 _= *αI*_02 _- *cR*_2 _- *R*_2_

R˙
 MathType@MTEF@5@5@+=feaafiart1ev1aaatCvAUfKttLearuWrP9MDH5MBPbIqV92AaeXatLxBI9gBaebbnrfifHhDYfgasaacPC6xNi=xI8qiVKYPFjYdHaVhbbf9v8qqaqFr0xc9vqFj0dXdbba91qpepeI8k8fiI+fsY=rqGqVepae9pg0db9vqaiVgFr0xfr=xfr=xc9adbaqaaeGacaGaaiaabeqaaeqabiWaaaGcbaGafmOuaiLbaiaaaaa@2D57@_3 _= *α*(*I*_12 _+ *I*_21_) - *cR*_3 _- *R*_3_.

where the dots over the host types denote derivatives with respect to time.

We express the dynamics in Eqs. (2–12) in terms of nondimensional quantities. The sum of all host types adds to one; the quantity of each host type measures the fraction of the total population. Each nondimensional time unit, *τ*, measures the average lifespan of a host in the absence of infection. For example, if the death rate per year is *φ*, then *τ *= *φt *is the number of host lifespans in *t *years, each lifespan of length 1/*φ *years. We sometimes refer to one lifespan, *τ *= 1, as a "generation."

To keep the sum of the host types constant at one, the first term on the right side of Eq. (2) provides an influx of newborn hosts at a rate that replaces all of the hosts that die over the course of an average lifespan. Equivalently, we may consider the influx into the naive class, *S*_0_, comprised of hosts that either die or lose their immunological memory. Those individuals who die are replaced with naive newborns; those individuals who lose their immunological memory also flow into the naive class, *S*_0_. We will use the terms "death" and "lifespan" to cover this broader interpretation that includes the decay of immunological memory.

By convention, we assume that pathogen type 1 has a transmission success greater than or equal to that of type 2. When type 1 can infect a host, its transmission rate is *β*, and when pathogen type 2 can infect a host, its transmission rate is *β *- *δ *= *β*(1 - *δ*/*β*), where *δ*/*β *is the transmission discount or cost for pathogen type 2 relative to type 1.

One additional factor may influence transmission. The naive class, *S*_0_, often corresponds to newborns or younger individuals who have not yet been exposed to infection, whereas the classes with memory of prior exposure often correspond to older individuals who have been previously infected. Transmission may occur more rapidly between newborns and younger individuals than between older individuals: the younger classes may aggregate more often in schools or play, or may be inherently weaker and more prone to infection.

We can account for relatively slower transmission among the exposed classes by reducing the transmission of new infections to those who carry memory of past infections. In particular, we weight transmission to susceptibles who have memory of prior infection by *γ *≤ 1.

The terms *αI*_*jk *_flow infected hosts into *R*_*j*+*k*_, that is, flow hosts who recover from infection at a rate *α *and become temporarily and nonspecifically immune to all pathogen types, with prior infection history *j *+ *k*. The terms *cR*_*j *_flow recovered and temporarily immune individuals with memory profile *j *into the susceptible class, where *c *sets the rate at which hosts lose short-term nonspecific immunity. The final term of each equation flows hosts out that class by death or by decay of memory and into class *S*_0_, where each increment of host generation time, d*τ*, corresponds to a fraction d*τ *of hosts who move from their current class into the naive class, *S*_0_.

To study invasion of a rare type, we first set the abundance of the rare variant (type 2) to zero and solve for the equilibrium state of the host population containing only the candidate dominant type (type 1), yielding

S0∗=1+αβ
 MathType@MTEF@5@5@+=feaafiart1ev1aaatCvAUfKttLearuWrP9MDH5MBPbIqV92AaeXatLxBI9gBaebbnrfifHhDYfgasaacPC6xNi=xI8qiVKYPFjYdHaVhbbf9v8qqaqFr0xc9vqFj0dXdbba91qpepeI8k8fiI+fsY=rqGqVepae9pg0db9vqaiVgFr0xfr=xfr=xc9adbaqaaeGacaGaaiaabeqaaeqabiWaaaGcbaqcfaOaem4uam1aa0baaeaacqaIWaamaeaacqGHxiIkaaGccqGH9aqpjuaGdaWcaaqaaiabigdaXiabgUcaRGGaciab=f7aHbqaaiab=j7aIbaaaaa@369F@

I01∗=11+α−1β
 MathType@MTEF@5@5@+=feaafiart1ev1aaatCvAUfKttLearuWrP9MDH5MBPbIqV92AaeXatLxBI9gBaebbnrfifHhDYfgasaacPC6xNi=xI8qiVKYPFjYdHaVhbbf9v8qqaqFr0xc9vqFj0dXdbba91qpepeI8k8fiI+fsY=rqGqVepae9pg0db9vqaiVgFr0xfr=xfr=xc9adbaqaaeGacaGaaiaabeqaaeqabiWaaaGcbaqcfaOaemysaK0aa0baaeaacqaIWaamcqaIXaqmaeaacqGHxiIkaaGccqGH9aqpjuaGdaWcaaqaaiabigdaXaqaaiabigdaXiabgUcaRGGaciab=f7aHbaacqGHsisldaWcaaqaaiabigdaXaqaaiab=j7aIbaaaaa@3A58@

R1∗=(α1+c)I01∗
 MathType@MTEF@5@5@+=feaafiart1ev1aaatCvAUfKttLearuWrP9MDH5MBPbIqV92AaeXatLxBI9gBaebbnrfifHhDYfgasaacPC6xNi=xI8qiVKYPFjYdHaVhbbf9v8qqaqFr0xc9vqFj0dXdbba91qpepeI8k8fiI+fsY=rqGqVepae9pg0db9vqaiVgFr0xfr=xfr=xc9adbaqaaeGacaGaaiaabeqaaeqabiWaaaGcbaqcfaOaemOuai1aa0baaeaacqaIXaqmaeaacqGHxiIkaaGccqGH9aqpcqGGOaakjuaGdaWcaaqaaGGaciab=f7aHbqaaiabigdaXiabgUcaRiabdogaJbaacqGGPaqkcqWGjbqsdaqhaaqaaiabicdaWiabigdaXaqaaiabgEHiQaaaaaa@3C0E@

S1∗=cR1∗.
 MathType@MTEF@5@5@+=feaafiart1ev1aaatCvAUfKttLearuWrP9MDH5MBPbIqV92AaeXatLxBI9gBaebbnrfifHhDYfgasaacPC6xNi=xI8qiVKYPFjYdHaVhbbf9v8qqaqFr0xc9vqFj0dXdbba91qpepeI8k8fiI+fsY=rqGqVepae9pg0db9vqaiVgFr0xfr=xfr=xc9adbaqaaeGacaGaaiaabeqaaeqabiWaaaGcbaGaem4uam1aa0baaSqaaiabigdaXaqaaiabgEHiQaaakiabg2da9iabdogaJjabdkfasnaaDaaaleaacqaIXaqmaeaacqGHxiIkaaGccqGGUaGlaaa@35E2@

We introduce a rare infection of the second variant (type 2) by setting the total fraction of hosts infected by the rare variant to a small value, *I*_·2 _= *I*_02 _+ *I*_12 _= *ε*. The rare type increases from an initially low level when I˙
 MathType@MTEF@5@5@+=feaafiart1ev1aaatCvAUfKttLearuWrP9MDH5MBPbIqV92AaeXatLxBI9gBaebbnrfifHhDYfgasaacPC6xNi=xI8qiVKYPFjYdHaVhbbf9v8qqaqFr0xc9vqFj0dXdbba91qpepeI8k8fiI+fsY=rqGqVepae9pg0db9vqaiVgFr0xfr=xfr=xc9adbaqaaeGacaGaaiaabeqaaeqabiWaaaGcbaqcfaOafmysaKKbaiaaaaa@2DD3@_·2 _> 0, which, from Eqs. (7,8), requires that

(β−δ)(S0∗+γS1∗)−α−1>0,
 MathType@MTEF@5@5@+=feaafiart1ev1aaatCvAUfKttLearuWrP9MDH5MBPbIqV92AaeXatLxBI9gBaebbnrfifHhDYfgasaacPC6xNi=xI8qiVKYPFjYdHaVhbbf9v8qqaqFr0xc9vqFj0dXdbba91qpepeI8k8fiI+fsY=rqGqVepae9pg0db9vqaiVgFr0xfr=xfr=xc9adbaqaaeGacaGaaiaabeqaaeqabiWaaaGcbaGaeiikaGccciGae8NSdiMaeyOeI0Iae8hTdqMaeiykaKIaeiikaGIaem4uam1aa0baaSqaaiabicdaWaqaaiabgEHiQaaakiabgUcaRiab=n7aNjabdofatnaaDaaaleaacqaIXaqmaeaacqGHxiIkaaGccqGGPaqkcqGHsislcqWFXoqycqGHsislcqaIXaqmcqGH+aGpcqaIWaamcqGGSaalaaa@4400@

which can be rearranged as

γS1∗S0∗+γS1∗>δβ,
 MathType@MTEF@5@5@+=feaafiart1ev1aaatCvAUfKttLearuWrP9MDH5MBPbIqV92AaeXatLxBI9gBaebbnrfifHhDYfgasaacPC6xNi=xI8qiVKYPFjYdHaVhbbf9v8qqaqFr0xc9vqFj0dXdbba91qpepeI8k8fiI+fsY=rqGqVepae9pg0db9vqaiVgFr0xfr=xfr=xc9adbaqaaeGacaGaaiaabeqaaeqabiWaaaGcbaqcfa4aaSaaaeaaiiGacqWFZoWzcqWGtbWudaqhaaqaaiabigdaXaqaaiabgEHiQaaaaeaacqWGtbWudaqhaaqaaiabicdaWaqaaiabgEHiQaaacqGHRaWkcqWFZoWzcqWGtbWudaqhaaqaaiabigdaXaqaaiabgEHiQaaaaaGaeyOpa4ZaaSaaaeaacqWF0oazaeaacqWFYoGyaaGaeiilaWcaaa@3FB3@

as given in Eq. (1) of the text.

### Age-related effects on transmission

In the dynamical equations of the previous section, pathogens transmit into hosts with memory of prior infection at a rate reduced by *γ*. In the mathematical epidemiology literature, the normal interpretation of *γ *would be cross reactivity of immune memory against the alternative antigenic variant (literature summarized in [19]).

We use *γ *differently in this paper. We start with the fact that older individuals almost always have a higher probability of prior infection than do younger individuals. In order to discount the transmission into older individuals relative to younger individuals, we simply reduce transmission into those with prior infection relative to those without prior infection. Our method provides a very simple approach, because we already must distinguish between different classes based on prior infection history, and so our age weighting does not require additional classes of hosts based on age, each class with further parameters that determine transmission properties. A detailed model of age structure, with additional classes for each age, would provide a more complete analysis of age-related effects, but at the cost of much complexity and of numerous parameters for which we do not have empirical estimates and for which the additional analytical detail provides no insight in relation to our fundamental question in this paper. That question is: Can age-related effects have a significant influence on antigenic variation?

The disadvantage of our approach is that the value of *γ *must depend on the other parameters. For example, as transmission, *β*, increases, the age distribution of infected and uninfected individuals changes, and so, holding all other parameters constant, *γ *must shift to account for the change in infection across age classes. At first glance, this dependence of *γ *on the other parameters may seem to be a problem for our method – in an ideal analysis, one would like parameters to be truly independent controls that one could change one at a time without altering the other parameters. However, such an ideal cannot exist.

Transmission, *β*, may often depend on the rate of clearance in the host, *α*, by coupling of those life history traits in the pathogen. Changes in transmission and clearance may often affect recruitment and population dynamics of the host population, and, in turn, external ecological changes of the host population must very often affect transmission and clearance.

In short, it is clearly naive to pretend that any parameter represents an independent control. For example, transmission is frequently treated as a independent parameter in mathematical epidemiology, yet transmission is likely to be the parameter most sensitive to changes in numerous other conditions. Making a model requires that one choose, by assertion, which factors are treated as independent parameters and which factors are treated as dependent variables. The choice depends on the purpose of the analysis and on expediency.

In our case, the simple control of age-related effects by *γ *provides the easiest way to see how such factors come into play in the analysis of antigenic variation. If our view proves to be useful, later models may explore the full age-structure analysis. But first, it pays to make the biological problem as transparent as possible.

## Authors' contributions

SAF and RMB jointly developed the concepts and background for the project. SAF wrote the first draft; SAF and RMB jointly rewrote and edited all sections. All authors read and approved the final manuscript.
